# American Medical Association

**Published:** 1865-08

**Authors:** Wm. B. Atkinson

**Affiliations:** Perm. Sec’y, Philadelphia


### American Medical Association.

Members who desire copies of the Transactions for 1865, must forward their subscriptions, ($3,) immediately, as the edition will be limited to the number required at the time of going to press.

WM. B. ATKINSON,
Perm. Sec'y, Philadelphia.

